# A structured mixed method process evaluation of a randomized controlled trial of Individual Placement and Support (IPS)

**DOI:** 10.1186/s43058-020-00083-9

**Published:** 2020-10-30

**Authors:** Tonje Fyhn, Kari Ludvigsen, Silje E. Reme, Frederieke Schaafsma

**Affiliations:** 1 NORCE Norwegian Research Centre, Postboks 7810, 5020 Bergen, Norway; 2 Department of Pedagogy, Religion and Social Studies, Western Norway University of Applied Sciences, Inndalsveien 28, 5063 Bergen, Norway; 3 Department of Psychology, University of Oslo, Forskningsveien 3A, 0373 Oslo, Norway; 4 Department of Public and Occupational Health, Amsterdam University Medical Centers, Amsterdam Public Health Research Institute, PO Box 7057, Amsterdam, 1007 MB The Netherlands

**Keywords:** IPS, Process evaluation, Recovery, Psychiatric disorders, Mental health services

## Abstract

**Background:**

Individual Placement and Support (IPS) is an evidence-based work rehabilitation program helping people with moderate to severe mental illness to obtain ordinary employment. Although IPS has proven superior to other work rehabilitation programs, in many studies, the majority of the participants remain unemployed. Structured process evaluations of IPS that use mixed methods are scarce, although they could identify implementation aspects that may enhance its effect. The aim of the current study is to assess reach, fidelity, and identify barriers and facilitators to implement IPS.

**Methods:**

The process evaluation was conducted alongside a randomized controlled trial including six IPS centers, comparing IPS with treatment as usual in a population of patients in treatment for moderate to severe mental illness. Mixed methods were used in the process evaluation, including focus group interviews with service providers, individual interviews and survey data from participants, and fidelity reviews using the validated IPS Fidelity Scale.

**Results:**

The intervention reached the intended target group. All centers reached fair to good fidelity according to the IPS Fidelity Scale within the project period (range 97–109, SD 8.1) (see Table 5). Certain fidelity items indicated implementation issues related to employer contact, community-based services, and integration with health services. Survey data showed that less than half of the participants regarded their illness as a barrier for participating in IPS and that freedom of disclosure was important. Participant interviews gave further insight into the role of the IPS specialist, emphasizing their availability and consistent job focus.

**Conclusions:**

Indications of implementation challenges across centers during the first year suggest special attention should be given to these aspects in an early phase to ensure higher fidelity from the start and thus enhance the effectiveness of IPS. The IPS specialist played an important role for participants and was described as positive, pushing in a positive way, and encouraging. More knowledge on the characteristics of successful IPS specialists could further enhance the effectiveness of the intervention.

**Trial registration:**

The study was registered on clinicaltrials.gov prior to the inclusion period (reg.no: NCT01964092, registered 17/07/2013).

**Supplementary information:**

**Supplementary information** accompanies this paper at 10.1186/s43058-020-00083-9.

Contributions to the literature

- The study describes a structured process evaluation of IPS using concepts from the implementation literature, which is lacking in previous IPS studies.
- The study uses mixed methods to gain a thorough understanding of barriers and facilitators to implementation and participation.
- The study supports the recovery notion that people with severe mental illness who are motivated to find employment should be offered help to achieve this.
- The study suggests a need for weighting IPS fidelity items according to their association with employment outcomes.

## Background

Mental disorders represent a significant barrier to employment [1]. The WHO’s “Mental Health Action Plan 2013-2020” advices the use of so-called multisectoral approaches to treatment, characterized by coordinated services to ensure not only basic health treatment, but also access to employment [2]. One such multisectoral approach is the vocational service Individual Placement and Support (IPS). IPS is an evidence-based approach helping individuals with severe mental illness to obtain ordinary employment [3]. It is based on a recovery approach to mental illness, emphasizing consumer-orientation, social support, and integration of services [4]. It is based on eight principles: Seeking competitive employment, rapid job search, systematic job development, integrated services, benefits planning, no exclusion, time-unlimited support, and participant preferences [5]. Participants are assigned an IPS specialist who provides face-to-face counseling, who is trained in the method, and who helps them get in touch with a potential employer within 30 days. The job search is guided by the participant’s preferences. The IPS specialist provides time-unlimited supports and follows up missed appointments with participants. The supports continue as needed after obtaining employment. The IPS specialist is integrated in participants’ health treatment team, in order to ensure coordinated services that facilitate work participation. The IPS specialist should spend 65% or more of their time outside the office, to ensure active follow-up of participants and face-to-face networking with employers in the community.

The IPS principles are operationalized in an implementation manual [6], which describes in detail the resources and preparations needed at different levels of the involved institutions to successfully implement the intervention. The principles are quantified in the validated IPS Fidelity Scale, which measures adherence to the method across different cultural contexts [7]. IPS programs obtaining high scores on this scale, as measured by evaluators, obtain higher shares of employment in ordinary jobs [8–10]. Through several experimental studies, the IPS method has proved more effective than traditional employment services, providing a robust empirical base for implementation across a wide variety of cultural contexts [11].

Although IPS has consistently proved effective, the majority of participants do not obtain employment [12–17]. Perhaps more thorough process evaluations or implementation studies could shed light on specific barriers and facilitators to be targeted in order to enhance employment outcomes. Process evaluations aim to improve the external validity of the outcome evaluation [18] and should describe what components of a given intervention are effective, for whom, and under what conditions [19, 20]. However, structured process evaluations of IPS are scarce. A recent review on implementation studies of supported employment revealed that the investigation of implementation issues take widely different approaches, from reflections based on anecdotes to semi-structured interviews and surveys [21]. The lack of common approaches makes it difficult to get an overview of implementation challenges that are generic across contexts, or specific to certain contexts. Such knowledge could enhance future IPS implementation efforts, particularly for piloting IPS for new target groups, such as patients with chronic pain, marginalized young people, and refugees [22, 23]. The process evaluation in the current study focused on assessing reach, fidelity of intervention delivery, and exploring barriers and facilitators to implementation and participation. These implementation measures were selected in accordance with the specifications in the governmental commission that initiated the study, and with recommendations found in Linnan and Steckler’s framework for conceptualizing process evaluations of public health interventions and research [19]. These authors define reach as the extent to which the intended target group actually participates in the intervention, and fidelity is defined as the extent to which the intervention was carried out as planned. Barriers and facilitators are defined in the current study as factors that obstruct or enable implementation of evidence-based practices [24].

The process evaluation was conducted alongside the randomized controlled trial of IPS in Norway [15, 25], and its function was to complement the results from the RCT, as well as enhance the effect of future IPS implementation efforts [15]. The aim of the current study is to answer the following research questions:

- Was the target group reached?
- What are barriers and facilitators to implementation as indicated by fidelity reviews and focus group interviews with service providers?
- What are barriers and facilitators to participation as indicated by follow-up surveys and individual interviews with participants?

## Methods

The process evaluation used mixed methods in the effort to identify barriers and facilitators to implement IPS, and the interplay between intervention components [26, 27]. Its methodological structure is QUAN + qual, meaning that quantitative and qualitative data was collected simultaneously, but the starting point for the analyses was the quantitative data [28].

### About the IPS trial

The IPS trial included an outcome evaluation, a process evaluation, and a cost/benefit analysis [25]. Participants were randomized to an intervention group receiving IPS in addition to treatment as usual (TAU), or to a control group receiving only TAU. Randomization was stratified by each pilot center. Results from the outcome evaluation showed that IPS was more effective than TAU, and the intervention group also showed improvements on the secondary outcomes of self-reported health and depressive symptoms, quality of life, and subjective health complaints [15]. The cost-benefit analysis showed that the intervention was not financially sustainable in the short term, but is likely to achieve this within a few years if the employment rate is sustained (ibid).

### Process measures

The process evaluation was designed as a summative, and not a formative, evaluation, meaning that its purpose was to generate knowledge about the implementation process of a standardized intervention [26]. An overview of the process measures is provided in Table 1.

**Table 1:** Data sources of the selected process measures at participant and service provider level

Process measure	Data source
*Participant level*	
Reach/population characteristics	Baseline survey and Mini-National Neuropsychiatric Interviews (M.I.N.I)
Barriers and facilitators	6-month follow-up survey, individual interviews
*Service provider level*	
Barriers and facilitators	Focus group interviews with IPS specialists, and fidelity reviews from each center
Fidelity	IPS Fidelity Scale (IPS-25)

*Reach* describes whether the study population corresponded to the pre-defined target population for the intervention. *Barriers and facilitators* aim to identify problems as well as helpful factors in the implementation of and participation in the intervention, as reported by IPS specialists and intervention participants. *Fidelity* measures adherence to the IPS method from service providers, by using the IPS Fidelity Scale [9].

### Study population

Data was collected from two populations: participants in the intervention group of the RCT and service providers.

#### Intervention participants

A total of 227 participants were randomized to the intervention group at inclusion, and 96 of these returned the 6-month follow-up questionnaire. Inclusion criteria for participants in the RCT were being in treatment for moderate to severe mental illness, having a desire to work, and understanding Norwegian well enough to respond to questionnaires. There were no significant differences between the intervention and control groups at baseline [15].

#### Service providers

Data from service providers was collected through fidelity reviews from each center and focus group interviews with the IPS specialist team at each center. Two focus groups consisted of three informants, and four groups consisted of five informants.

### Data collection

#### Reach

Reach was measured through quantitative baseline survey data as well as results from the Mini-International Neuropsychiatric Interview (M.I.N.I), a short, structured diagnostic interview [29] which was conducted at inclusion.

#### Barriers and facilitators to participate in IPS

Data was collected through individual interviews with intervention participants, as well as items in the 6-month follow-up questionnaire. Informants were recruited based on their written consent in the 6-month follow-up questionnaire and were randomly selected from a computer-generated list of participants who reported to be “less satisfied” to “very satisfied” as indicated in the questionnaire. Twelve participants agreed to be interviewed. The interviews followed a semi-structured interview guide and lasted up to 20 min. Interviews were conducted by author KL and a research assistant. All participants who were interviewed signed an informed and written consent for this particular sub-study. Items included in the 6-month follow-up survey were constructed for the current study and were not validated. Items included a list of statements concerning six proposed barriers and six proposed facilitators of participation (yes/no), which articulated aspects of IPS that differed from ordinary work rehabilitation programs, or other aspects of the intervention that could possibly obstruct or enable participation for this target group. An open-ended response category was added under each list. Satisfaction with the IPS specialist was measured through the item “How satisfied are you with your IPS specialist?.” Perceived usefulness was measured through the question “How useful has it been for you to participate in IPS?” Response categories for each question ranged from 1 = very dissatisfied/not useful at all to 5 = very satisfied/useful.

#### Barriers and facilitators to implement IPS

Data was collected through focus group interviews with IPS teams, and fidelity reviews. IPS specialists and team leaders were asked to participate in interviews as a team, which were conducted by author KL at all six IPS centers. The interviews followed a semi-structured interview guide and lasted for approximately 1.5 h. The interview guide was developed by drawing on experiences from a previous project with high resemblance to the current and by assessing specifications in the governmental commission. Interviews were recorded on tape and transcribed before analyses. All informants were informed of the purpose of the interviews, that participation was voluntary, and of their right to withdraw at any time.

The fidelity reviews were carried out at each center approximately 1 year into the study period by a trained evaluator team who followed instructions in the IPS Fidelity Review Manual [6]. Low overall fidelity scores indicate implementation challenges, and low scores on single items indicate what those challenges are. The scrutiny of the IPS Fidelity scale enables a more fine-grained examination of possible implementation issues than general scales of program adherence. Fidelity measurements are therefore included as indicators of barriers and facilitators to implementation, complemented by focus group interview data.

### Data analysis

To investigate reach, descriptive analyses were conducted on baseline data and M.I.NI. results, using SPSS 25 and Excel (2017).

Quantitative and qualitative data on barriers and facilitators were collated through the following steps, performed separately for each participant group: (1) quantitative data was analyzed, (2) qualitative data was analyzed, and (3) survey items or fidelity items that were particularly low or high were used as an indicator to select themes from the interviews that could further describe these issues. Lists of themes derived from the interviews are included in the supplementary material.

#### Barriers and facilitators to participate in IPS

Descriptive analyses were conducted on survey data using SPSS 25. To identify barriers and facilitators, results were interpreted by looking for frequently occurring responses in the lists of barriers and facilitators, or unevenly distributed responses to the satisfaction/usefulness items.

Qualitative data was analyzed through a deductive approach, using thematic analysis as described by Boyatzis [30] and Joffe and Yardley [31]. A coding scheme was constructed based on topics in the interview guide and expanded or revised according to recurring topics in the data. The interviews were studied repeatedly and categorized according to this scheme. The analysis focused on manifest content rather than latent phenomena [31], to facilitate bridging of the qualitative and the quantitative data [30]. The unit of analysis was sentences or shorter paragraphs where several sentences described the same topic. Author KL conducted the first round of analyses, and TF supplied and adjusted themes in the second round of analyses. The two authors agreed on the final themes to be included in the study.

Quantitative and qualitative results were collated through the steps described above, using quantitative findings as a lens to select themes from the qualitative results. Some interview themes contained descriptions that were specific to the Norwegian context; others lacked substance and coherence. These were not included in the subsequent analysis, but were described in the final report to the study commissioner [32].

#### Barriers and facilitators to implementation

The same analytic approach was used for the focus group interviews.

Fidelity reviews were summarized in an Excel table where mean, minimum, and maximum scores and disparities between high-performing and less-performing centers were calculated. Items with particularly low or high scores across centers 1 year into the study period were assumed to indicate implementation barriers or facilitators.

## Results

### Reach

The target group for the trial, as defined in the governmental commission of the study, were people in treatment for moderate to severe mental illness in secondary care. The diagnostic screening of participants at inclusion showed that 51% of the participants suffered from severe mental illness (psychosis or bipolar disorder) and 49% fulfilled criteria for moderate mental illness (primarily affective disorders). This indicates that the study population corresponds to the pre-defined target group.

An overview of study population characteristics is provided in Table 2.

**Table 2:** Characteristics of the intervention group at baseline

Variable	%	***n***	Mean	SD
Age		185	35	10.7
Gender (female)	51	185		
Higher education	24	182		
Reading/writing disabilities	15	182		
Years of employment experience		154	7.4	7
Health-related quality of life (EQ-VAS; 0-100)		172	58	18.3
Functioning (WHODAS; 0-48)		179	22	14
Previously experienced violence	48	180		
Previously involuntarily committed	31	173		
Years with mental health complaints		141	10.8	9.2
Psychiatric diagnoses (M.I.N.I. Interviews)				
Recurrent depression	49	140		
Psychosis	41	142		
Anxiety	60	141		
Substance addiction	15	165		
Severe mental illness	51.4	146		
Moderate mental illness	48.6	146		

The study population was relatively young (*x* = 35, SD 10.7) and education level was low. Nearly half of the participants had experienced violence, and one third had been involuntarily committed to a psychiatric hospital. Mean of previous years worked in main occupation was 7 (SD 7). The mean rating of health-related quality of life, measured by the EQ-5D visual analogue scale, was 58 (SD 18.3).

### Barriers and facilitators to participate in IPS

Table 3 shows results from the barriers and facilitators to participation lists. Open-ended response categories were provided, but they did not generate additional barriers or facilitators.

**Table 3:** Percentage and number of respondents agreeing to the statements about facilitators and barriers

Facilitators for participation	Yes	***n***	Barriers for participation	Yes	***n***
It was helpful that progress was quicker than other vocational services	65%	77	Progress was made too quickly	13%	76
Knowing the IPS specialist was available was helpful	92%	77	It was too time-consuming	9%	77
The action steps along the way were specific, and this was helpful	79%	76	I had challenges with my IPS specialist	8%	76
Freedom of disclosure was helpful	92%	76	My illness was a barrier	43%	77
The support plan I made with the IPS specialist when I got a job was helpful	52%	64	IPS was not what I expected	17%	77
The regular follow-up from the IPS specialist in the job search was helpful	79%	72	Getting to the different places (to meet employers or IPS specialist)	9%	76

Two of the most frequently cited facilitators among participants regarded the IPS specialist’s role: 94% of respondents agreed with the statement “Knowing that the IPS specialist was available for me was helpful,” while 81% agreed with the statement “The regular follow-up from the IPS specialist was helpful.”

Responding to a separate item regarding the IPS specialist, 78% reported to be satisfied, while 13% reported to be dissatisfied. Nine percent reported to be neither dissatisfied nor satisfied (*n* = 78; *x* = 4 SD 1.11).

All over, participants were very happy with the role of the IPS specialist. Participant interviews gave further insight into the IPS specialist’s role, emphasizing their availability, support, and consistent job focus. When talking about availability, informants emphasized that the IPS specialist was quick to respond and to express their availability:

She is really good, really efficient. Supportive, calls and asks me to call back, to call on her spare time. If it’s a good time it’s a good time, if it’s not a good time she calls me back up again. It’s been really nice. (Informant 2)

I think he’s been really available, because even if he doesn’t answer my call immediately, he calls me back up, he is always there for me if anything comes up. So yes…I feel I have received very, very good follow-up from him. So I am very happy. (Informant 8)

Some participants said that the IPS specialist “pushed” them to keep going with the job search, and some had been confronted with their lack of motivation. This resulted in taking a more active role in the job search:

It wasn’t a threat, but they said we can’t help you if you’re not interested. They deserved an honest answer to that. It was the best question I could have received, instead of them saying ‘We’re not wasting time on this...’ I woke up. (Informant 10)

It has been positive for me to start working, yes. But I do feel there is a small pressure and that I have to push myself to say yes to working. I am supposed to start working and not sit at home. And I did get a job, so maybe it’s good that they push a little. (Informant 2)

One participant described how being listened to in the process was important:

If he has come with a job suggestion and I have said that this is not for me, because I will not function well there, he has just put it away immediately, he is very accommodating like that. (Informant 4)

There were a few exceptions to the positive descriptions of the IPS specialist’s role. Two of the informants who were less satisfied with the intervention described the interaction with the IPS specialist as challenging.

Another facilitator for participation indicated by survey data was the freedom to disclose or not: 93% of respondents agreed that “Being able to choose whether or not to be open about my illness” was helpful. The interviews indicated that for some participants, choosing to disclose expanded the possibilities for practical help in the job search:

Yes, it’s very nice because the IPS specialist can call around for me, I have anxiety about talking on the phone sometimes. And she is with me in the conversations with employers so that I understand what is being said, and she can also inquire about salary. (Informant 2)

One participant reflected on the positive aspects of disclosing to potential employers:

It might be that some employers think that they want to support it because it is kind of a good cause to help people get a job that maybe have a history of illness or have had problems, and because of that they can get a job. (Informant 7)

Most of the proposed barriers were not supported by participants. However, the most agreed-upon statement was “My illness was a barrier” (46%, *n* = 95). Considering the target group of the intervention, this number is not particularly high. Informants described illness factors mostly in the context of having the IPS follow-up tailored to their health condition. One in six participants agreed to the statement “IPS was not what I expected” (17%, *n* = 77). Examples of this were found in the interviews. While some participants were positively surprised by the intervention, others described being disappointed due to high expectations.

I was promised employment within 6 weeks, and now I have waited for 13-14 months (…). I had expectations about follow-up from IPS specialist and close cooperation between my doctor, the District Psychiatric Center, and a permanent position with full salary. And none of it has happened. (Informant 6)

One informant stated that the follow-up was simply different from what he had expected regarding his own involvement:

Uuuhm, but the only expectation I did have that turned out not to be correct was that I kind of thought they had some sort of obligation to help me find a job so I didn’t have to do such an effort myself. But that was totally wrong. (…) It’s not like I can nag them and say ‘Hey, find me a job’, it’s more like they come alongside and back me up on the things I manage to do. (Informant 5)

Two items measuring satisfaction and perceived usefulness were included as indicators of barriers and facilitators to participation, as low scores on these measures could indicate poor quality in intervention delivery, and/or low engagement with the intervention among participants (Table 4). However, participants were overall satisfied with the intervention (*n* = 95; *x* = 3.95 SD 0.97) and also found it useful (*n* = 96; *x* = 3.96 SD 1.06).

**Table 4:** Participants’ satisfaction and perceived usefulness of the intervention

	%	***n***
**General satisfaction (*n*** **= 78)**		
Dissatisfied	1	1
Not very satisfied	9	7
A little satisfied	18	14
Pretty satisfied	37	29
Very satisfied	35	27
**Usefulness (*n*** **= 78)**		
Not useful at all	4	3
Not very useful	4	3
A little useful	24	19
Pretty useful	30	23
Very useful	39	30

Participant interviews provided further insight about this. Informants said that IPS had made them aware of their own competence and preferences. Most informants reported that IPS had increased the frequency of sending applications and that they had learned more about going to job interviews. They emphasized that the focus on employment had been central in the follow-up:

I haven’t got a job offer, but now I apply for jobs in a different way. I have been on many interviews, so that has improved as a result of this follow-up (…). I learned how to write an application, about motivation, qualities…. (Informant 11)

The first thing we did was to go over what kind of jobs I wanted, then we got my CV sorted out, how to write an application, and have everything ready for sending the application. (…) And then we went out into the job market. We went step by step, one thing after another, in the right order. (Informant 10)

### Barriers and facilitators to implement IPS

Barriers and facilitators to implementation were examined using the IPS Fidelity Scale and focus group interviews with IPS teams. Results are presented in Table 5. All six centers had reached fair or good fidelity at this point, with scores ranging between 97 (see Table 5) and 109 (median 99.5), with 125 as the highest attainable score, and 74 as the cut-off score for being IPS. Single items were rated on a range from 1 to 5, where item scores 1–3 indicate no or poor implementation.

**Table 5:** Center scores on each item, mean scores of centers, mean of lowest and highest performing centers, and total fidelity score for each center

IPS Fidelity Scale (IPS-25)	Mean all centers	Less performing centers				High-performing centers			
		Center 1	Center 2	Center 4	Mean	Center 3	Center 5	Center 6	Mean
Case load size	5.0	5	5	5	5.0	5	5	5	5.0
Exclusively vocational services	4.5	5	5	2	4.0	5	5	5	5.0
Vocational generalists	4.6	4	5	5	4.7	5	4	5	4.5
Integration of IPS with treatment team	3.6	5	2	2	3.0	4	5	4	4.2
IPS team contact with treatment team	2.5	2	3	3	2.7	3	1	3	2.3
State vocational rehabilitation agency is actively involved	4.3	3	5	5	4.3	5	4	4	4.3
IPS team forms a vocational unit	4.8	5	5	4	4.7	5	5	5	4.8
Supervisory role of IPS team leader	3.6	4	3	3	3.3	5	3	4	3.8
Zero exclusion of clients	3.3	3	4	3	3.3	3	3	4	3.3
Agency focus on work	2.6	2	3	3	2.7	3	2	3	2.5
Agency leadership support	3.5	5	4	3	4.0	3	2	4	3.0
Benefits counseling	4.8	4	5	5	4.7	5	5	5	5.0
Disclosure of disability to employers	4.8	5	5	5	5.0	5	4	5	4.5
Individualized assessment	4.7	5	5	5	5.0	4	4	5	4.3
Rapid search	3.7	3	5	3	3.7	3	3	5	3.7
Individualized job search	4.8	5	5	5	5.0	5	4	5	4.7
Job development, frequency	1.8	1	2	1	1.3	3	1	3	2.2
Job development, quality	3.7	2	1	4	2.3	5	5	5	5.0
Occupational diversity	4.6	5	5	5	5.0	4	4	5	4.2
Employer diversity	4.9	5	5	5	5.0	5	5	5	4.8
Competitive jobs	3.6	4	5	1	3.3	5	3	4	3.8
Individualized supports	4.5	4	5	5	4.7	3	5	5	4.3
Time-unlimited supports	4.1	5	5	4	4.7	4	3	4	3.5
Community-based services	1.2	1	1	1	1.0	1	1	2	1.3
Assertive outreach to clients	3.9	5	5	2	4.0	4	3	5	3.8
Total fidelity score		97	103	89		102	89	109	

Items with low scores across centers were “Community-based services” (*x* = 1) and “Job development—frequency” (*x* = 2). To receive a top score on “Community-based services,” the IPS specialists must spend 65% or more of their time outside the office, following up participants in their local area. This should be seen in relation to the item “Job development—frequency,” indicating frequency of contact with employers in order to develop a broad employer network. The IPS specialists reported in the interviews that it had taken quite some time to develop and understand their role and that prioritizing tasks was demanding, as expressed in the following remarks:

What we always have to challenge ourselves on is the use of time, considering time spent on internal meetings, participant meetings and employer contact. To obtain the optimal allocation of resources is pretty challenging.

Being an IPS specialist is a pretty complex and difficult role, where you are a seller on the one hand, selling the best manpower there is, next you’re a facilitator, an IPS specialist, and you can sometimes have a therapeutic approach at times when the therapist is not there. So, it is a very difficult role, and it takes time to be secure in it.

Looking at the two items measuring “Integration with health services,” which is an important IPS principle, the mean score of the two items across centers is 3. According to the quality thresholds defined by the program developers, this is barely above the “Fair fidelity” threshold [9]. These items measure whether the IPS specialists are integrated in participant’s health treatment team, attend weekly meetings, and ensure focus on employment in coordinating services for the participant.

On the positive side, all six centers received top scores on the items “Caseload size” and “Employer diversity,” and nearly top score on “Disclosure of disability to employers.” “Caseload size” means that the caseload for each IPS specialist does not exceed 20 participants, in order to ensure close follow-up in all phases of the job search. “Employer diversity” refers to the diversity of workplaces where participants get jobs. It is used as an indicator of whether IPS specialists are following participants’ own preferences, and not only working within the limits of their existing employer network. Disclosure measures whether IPS specialists provide information to participants about pros and cons of disclosing about their illness to an employer.

The effect evaluation showed that three of the six pilot centers performed particularly well on employment outcomes [32], though there were no obvious reasons for this. The top three centers did, however, differ from the rest on two particular fidelity items. They averaged 1.3 points above the average of the less performing centers on the item “Integration of IPS with treatment team,” which is considered a crucial intervention component [3]. The challenges related to this topic were addressed frequently in the interviews and are illustrated by the following remark:

I think that it is the greatest success and the greatest challenge, that integration process (…), how we feel that they [treatment team] talk and feel concerning work.

However, the most striking difference was found between scores on “Job development—quality” (indicating quality of employer contact), where the top performing centers averaged 2.7 points above the average of the less performing centers. The issue of employer contact was addressed in the interviews, as exemplified in the following remarks:

…the job development part, that’s something that for most of us, and definitely for me, has been new and different, going out and being assertive both on the phone and in person (…) After a while I realized it’s been written in the manual the whole time, that we really need to have our main focus on job development. We’ve made some changes now this fall where we have set targets and try to reserve days and times to do that.

## Discussion

The results indicate that the target population for the intervention was reached and indicate certain barriers and facilitators to participate and to implement IPS.

### Reach

Participants’ characteristics correspond to the specification of the pre-defined target group. However, the original IPS target group is people with severe mental illness. No differences in employment outcomes between people with moderate vs severe mental illness were found in the outcome study [15]. This may indicate that IPS is effective also for an extended target group. Some interesting points can be drawn from the characteristics of the study population. First, although all participants are in treatment for moderate to severe mental illness, mean level of health-related quality of life is on the upper half of the scale. This shows the importance of subjective measures of health and well-being for this target group. It is also worth noticing that education level is relatively low, and the frequency of adverse events in the past (violence, having been involuntarily committed) is high in the population. In spite of these characteristics, the intervention proved effective on employment outcomes, and participants found it useful even though the majority did not obtain employment.

### Barriers and facilitators to participate in IPS

The role of the IPS specialist was perhaps the clearest facilitating factor emerging from both survey and interview data. Participants emphasized the availability of the IPS specialist, their attentiveness to participant preferences, and pushing participants to take steps out of the comfort zone. The consistent employment focus in the follow-up was important for participants’ motivation and learning. This is in line with a study identifying emotional support, practical assistance, and a client-centered approach as effective ingredients from the participant perspective [33]. An ethnographic study of IPS specialist skills identified efficiency, good collaboration with partners, and developing egalitarian relationships with participants as skills differentiating successful specialists from less successful ones [34]. The findings in the current study aligns well with these results, but particularly highlights the consumer-oriented, empowering approach of the IPS specialists.

Another important facilitator, found in participant data as well as in fidelity reports, was the freedom of disclosure. The participants seemed to value the support and information regarding this topic. A previous study has found this item to correlate positively with employment outcomes [35].

The facilitating factors discussed above align well with the values of recovery ideology, which has inspired the development of IPS [4]. Central ideals, such as empowerment and functioning in valued roles are reflected in participants’ reports, as well as being evident in the principles guiding the intervention.

This may partly explain why so many participants were very satisfied with the intervention and found it useful, despite the fact that at 18 months the majority (63%) had not obtained employment [15]. However, the outcome evaluation showed that intervention participants reported lower levels of self-reported psychological distress and somatic symptoms, and increased levels of functioning, quality of life, and well-being (ibid). Moreover, satisfaction and usefulness may reflect changes in participants’ orientation towards ordinary employment, and that they have gained useful knowledge and self-esteem in the job search process, as indicated by a recent meta-ethnographic review [36].

Few participants agreed to the barriers presented. Less than half of the respondents agreed that their illness was a barrier, which may be explained by the individual tailoring of the intervention, as expressed in participant interviews.

### Barriers and facilitators to implement IPS

The barriers to implementation as indicated by fidelity reviews and interview data represent untraditional approaches to providing vocational services and are all related to the role of the IPS specialist. *Providing community-based services* has been reported as a challenge in other IPS studies [37–39], as well as a facilitator for employment outcomes [40], and thus seems to be an important, but challenging component that may take some time to develop.

Succeeding with *job development* was also indicated by fidelity data as a challenging, but important component. Job development is a far more assertive approach than traditional employment services [41], which is characterized by using subsidized employment, sheltered employment, and work practice. The quality of job development efforts may indicate a make-or-break point for success [42, 43].

As for *integration with health services*, this is a core principle in IPS and indicated in the literature as crucial for successful implementation [3]. Barriers to integration seem to be rooted in structural barriers, cultural differences in institutions, and attitudes [36, 41, 44, 45].

Two facilitators are reflected in both populations in the current study, which also diverge from traditional follow-up. First, *disclosure* is often not a topic in traditional services, as the service provider usually makes the initial employer contact, directly or indirectly revealing participants’ health issues. Second, *the IPS specialist* has the capacity to provide individualized follow-up due to a smaller caseload than in traditional services. Furthermore, s/he acts as a mediating link between the job seeker and the employer, through job matching and by increasing access to a variety of jobs. These facilitators enable individualized tailoring of the follow-up, which is likely to enhance participants’ motivation and sense of autonomy, and thus may create sustainable workforce participation through a good job match.

### Strengths and limitations

Strengths of the study are its structured and mixed methods approach, with defined measures. This has enabled a thorough and multi-faceted evaluation of the intervention and enabled an integration of quantitative and qualitative findings. Compared to most other IPS evaluations, it provides richer data material from which to draw conclusions and identify areas for future research.

One limitation pertains to the validity of the fidelity reviews. It is recommended that reviewers are independent [46], which they were not in this study. However, this does not seem to be uncommon [13, 47–49]. Moreover, some IPS specialists questioned the training of the evaluators in the initial phase. Another limitation lies in the participant interviews, as far more satisfied participants than dissatisfied ones agreed to be contacted, leading to selection bias. Finally, the steps taken to collate the quantitative and qualitative results served the purpose of distilling large amounts of data; however, performing these steps with an inductive approach would likely have emphasized other parts of the data, which readers should keep in mind.

## Conclusions

Various facilitators and barriers to participate in and implement IPS were identified in the current study. For participants, the IPS specialist seemed to play a crucial role, as did the freedom to disclose or not. Barriers to participation were difficult to detect. One’s illness was not seen as a barrier for most participants, which strengthens the legitimacy of offering IPS to this population. Barriers to implement IPS included providing community-based services, employer contact, and integrating services, while facilitators to implementation included the fulfillment of the IPS specialist role, freedom of disclosure, and caseload size. Job development also seemed to be an important, but challenging component to implement. The novelty of the mentioned components in IPS may explain why they are challenging to implement, while clearly meeting a need among participants. As evidence on the effect of IPS increases, measures should be undertaken to enable a weighting of the different fidelity items [50], identifying crucial components of the intervention. This can facilitate more effective implementation of the intervention across contexts and possibly enhance its effect on employment outcomes.

## Supplementary information

**Additional file 1.** Qualitative themes.

## Data Availability

The datasets generated in the study are not publicly available due to restrictions imposed on data sharing by the Norwegian Social Science Data Services.

## Abbreviations

IPS: Individual Placement and Support
TAU: Treatment as usual
WHO: World Health Organization
