# Supplementary material for: A structured mixed method process evaluation of a randomized controlled trial of Individual Placement and Support (IPS)
Source: Implement Sci Commun. 2020 Oct 30;1:95. doi: 10.1186/s43058-020-00083-9 (PMC7599092; doi:10.1186/s43058-020-00083-9)
Supplement: Supplementary file 1 — Additional file 1. Qualitative themes. [file 43058_2020_83_MOESM1_ESM.docx]

| **Focus group interviews IPS specialists** | **Individual participant interviews** |
| --- | --- |
| IPS Specialist role development* | Motivation for and expectations towards the intervention* |
| Paid employment in ordinary work life* | Intervention's clear focus on work* |
| Written information about job services and work opportunities | Interaction with IPS specialist* |
| Focus on adults with severe mental illness | Engagement with intervention* |
| Sharing experiences between pilot centers | Perceived usefulness* |
| Extent of employer contact* | The job search process* |
| Intergrating job services and health treatment* | Individual adjustments made according to participants' health condition* |
| No exclusion of participants | Suggestions for improvement |
| Steep learning curve for pilots in initial phase |  |
| Challenges with directorate governance |  |
| Local variations in ownership of pilot center (Labour and Welfare Administration or Health directorate) |  |
| Variations in quality of IPS specialist training |  |

**Qualitative themes**

Themes derived from focus group interviews with IPS specialists and individual participant interviews (asterisks mark themes discussed in the article).
